# The stiffness‐controlled release of interleukin‐6 by cardiac fibroblasts is dependent on integrin α2β1

**DOI:** 10.1111/jcmm.15974

**Published:** 2020-10-30

**Authors:** Małgorzata Gałdyszyńska, Justyna Bobrowska, Małgorzata Lekka, Paulina Radwańska, Lucyna Piera, Jacek Szymański, Jacek Drobnik

**Affiliations:** ^1^ Laboratory of Connective Tissue Metabolism Department of Pathophysiology Medical University of Lodz Lodz Poland; ^2^ Institute of Nuclear Physics PAN Kraków Poland; ^3^ Central Scientific Laboratory Medical University of Lodz Lodz Poland

**Keywords:** heart, inflammation, integrin, interleukin, soft gel, stiff gel

## Abstract

Cardiac fibroblasts are able to sense the rigidity of their environment. The present study examines whether the stiffness of the substrate in cardiac fibroblast culture can influence the release of interleukin‐6 (IL‐6), interleukin‐11 (IL‐11) and soluble receptor of IL‐6 (sIL‐6R). It also examines the roles of integrin α2β1 activation and intracellular signalling in these processes. Cardiac fibroblasts were cultured on polyacrylamide gels and grafted to collagen, with an elasticity of *E* = 2.23 ± 0.8 kPa (soft gel) and *E* = 8.28 ± 1.06 kPa (stiff gel, measured by Atomic Force Microscope). Flow cytometry and ELISA demonstrated that the fibroblasts cultured on the soft gel demonstrated higher expression of the α2 integrin subunit and increased α2β1 integrin count and released higher levels of IL‐6 and sIL‐6R than those on the stiff gel. Substrate elasticity did not modify fibroblast IL‐11 content. The silencing of the α2 integrin subunit decreased the release of IL‐6. Similar effects were induced by TC‐I 15 (an α2β1 integrin inhibitor). The IL‐6 levels in the serum and heart were markedly lower in α2 integrin‐deficient mice B6.Cg‐Itga2^tm1.1Tkun/tm1.1Tkun^ than wild type. Inhibition of Src kinase by AZM 475271 modifies the IL‐6 level. sIL‐6R secretion is not dependent on α2β1 integrin. Conclusion: The elastic properties of the substrate influence the release of IL‐6 by cardiac fibroblasts, and this effect is dependent on α2β1 integrin and kinase Src activation.

## 1 INTRODUCTION

The behaviour of cells within a tissue is regulated not only by biochemical signals coming from the extracellular space but also by the physical nature of cell environment.^1^, ^2^ Two types of mechanosensitivity can be defined, this being the response of a cell to external physical forces: the first refers to the effect of an incident environmental force placed on the cell, while the other is associated with the response of the cell to the stiffness of the surrounding substrate.^1^ During heart fibrosis, the surroundings of the cardiac cells gradually stiffen.^1^, ^3^, ^4^, ^5^ Therefore, the increased rigidity of the extracellular space is not only the end‐point of fibrosis, but can also considered a factor regulating collagen accumulation within the tissue.^2^ Fibroblasts are able to sense the mechanical properties of the environment. Generated mechanical stimuli are initially recognized by extracellular collagen bound to integrins, following which the signal is transduced to effectors by a cellular pathway.^1^, ^2^, ^3^, ^4^, ^5^

Fibrous tissue is characterized by excessive synthesis and defective degradation of collagen, caused mainly by the action of activated cardiac fibroblasts. Such an accumulation of fibrous tissue has been implicated in increased rigidity of heart tissue and disturbances in heart rhythm associated with impaired electrical conduction in tissue.^3^, ^4^, ^5^ Cardiac fibrosis is typically accompanied by inflammation, which causes the secretion of pro‐inflammatory cytokines, including interleukin‐1β (IL‐1β), interleukin‐6 (IL‐6) and tumour necrosis factor α (TNF‐α). In addition to their pro‐inflammatory function, they may also regulate collagen accumulation.^3^, ^4^, ^5^, ^6^ Interestingly, in heart tissue, those cytokines are secreted by non‐immune cells such as cardiomyocytes and cardiac fibroblasts.^6^, ^7^

IL‐6 is well known to demonstrate profibrotic effects within the heart^8^, ^9^, ^10^ and is thought to potentially stimulate the TGF‐β signalling pathway.^11^ The final effect of IL‐6 is strongly influenced by the duration of its elevation, and as such, the presence of increased levels of IL‐6 released during inflammation following tissue injury may prevent cardiomyocyte apoptosis. However, chronic augmentation of IL‐6 has been found to lead to cardiac hypertrophy and heart fibrosis, and such elevation has been observed in blood samples taken from patients with various cardiovascular diseases.^12^, ^13^, ^14^ IL‐6 binds to a receptor complex consisting of specific IL‐6 binding protein (IL‐6R) and signal transducing molecule (gp130). Furthermore, the soluble form of IL‐6R (sIL‐6R) is generated by limited proteolysis of the membrane‐bound IL‐6R or by translation of spliced mRNA. The circulating IL‐6 and sIL‐6R complex activates signal transduction molecules and supports its activity. Expression of IL‐6R or sIL‐6R was observed in primary human fibroblast cultures derived from lungs^15^ and corneal fibroblasts^16^ and in fibroblast‐like cells derived from monkey kidney.^17^ The aim of the present study was to determine whether the mechanical properties of the cell environment may regulate the secretion of IL‐6, interleukin‐11 (IL‐11) from the IL‐6 family and soluble receptor of IL‐6 (sIL‐6R) by cardiac fibroblasts. It also examines whether integrin α2β1 and the kinases FAK and Src may be involved in regulation of cytokine release by fibroblasts. Recently published data indicate that IL‐6 may play a role in fibrosis in the cardiovascular system;^8^, ^9^, ^10^ however, its relationship with α2β1 integrin and the physical properties of the environment have not been determined. α2β1 integrin was selected for the present study because it belongs to the family of collagen‐binding integrin receptors. In addition, α2β1 integrin is known to be a mechanotransducer expressed on the cardiac fibroblast membrane.^18^, ^19^, ^20^

## 2 MATERIALS AND METHODS

### 2.1 Polyacrylamide gel preparation

The soft and stiff polyacrylamide gels were prepared to mimic the cellular environment with different mechanical properties. To produce the desired gels, 40% polyacrylamide (final concentration 8%) (BioRad) and 10 mmol/L HEPES pH 8.5 (Sigma‐Aldrich); the stiff gels were formed using 0.1% N,N'‐methylene‐bis‐acrylamide, while the soft gels used 0.06% N,N'‐methylene‐bis‐acrylamide (BioRad). The solutions were polymerized with 10% ammonium persulfate (Sigma‐Aldrich) and N,N,N',N'‐tetramethylenediamine (Sigma‐Aldrich) for about 30 minutes between sterile glass plates. After polymerization, the plates were separated, and 2.5‐cm‐diameter gel discs were cut and placed into 6‐well culture plates for further experiments.

The gels were activated by the addition of 0.5 mmol/L sulfo‐SANPAH (Sigma‐Aldrich) in solution containing 50 mmol/L HEPES pH 8.5 and 0.5% DMSO on the surface of each gel disc and then exposed to UV light for 30 minutes. The gels were washed three times with 50 mmol/L HEPES pH 8.5 (Sigma‐Aldrich). The gels were then coated with 100µg/ml solution of type I collagen (10 µg/cm^2^), (Sigma‐Aldrich) in 0.1 M acetic acid and incubated overnight at 4°C. Excess acetic acid was removed before use by washing with HBSS (Thermo Fisher Scientific).

### 2.2 Atomic force microscope

The elastic properties of the polyacrylamide gels were measured using atomic force microscope (Nanowizard 4, JPK Instruments) operating in force spectroscopy mode. Force curves, that is the relationship between cantilever deflection and relative sample position converted into force‐indentation curves, were measured within a scan area of 10 µm × 10 µm (100 curves per each scan area) at a load speed of 8 µm/s. Three independent polyacrylamide gel samples for each substrate stiffness were measured. On each sample, typically, 10 randomly selected scan areas were recorded, together with exemplary scan areas of 50 µm × 50 µm. The cantilevers used in the study were made of silicon nitride with a nominal spring constant of 0.02 N/m (OTR4, Bruker). To obtain information on the elastic modulus of the polyacrylamide gel samples, JPK software was used.

### 2.3 AFM‐based determination of the elastic (Young's) modulus

In AFM measurements, Young's modus was determined based on the comparison of the calibration curve recorded on a stiff, non‐deformable glass surface with the force curves were recorded on polyacrylamide gel samples as described in detail elsewhere.^21^, ^22^ Briefly, the Hertz‐Sneddon model was fitted to each force‐indentation curve to calculate the elastic modulus. The final Young's modulus was determined as mean ± standard deviation (SD) from all measured locations on polyacrylamide gel samples.

### 2.4 Cell culture

The study was conducted on an immortalized human cardiac fibroblast cell line obtained from ABM (Richmond, BC, Canada) and cultured in PriGrow IV medium (ABM) supplemented with 10% foetal bovine serum (FBS) (ABM), L‐glutamine (ABM), penicillin/streptomycin Solution (ABM), non‐essential amino acids (NEAA) (Sigma‐Aldrich), 5 µg/mL insulin (Thermo Fisher Scientific) and 50 µg/mL vitamin C (Sigma‐Aldrich) on the plates coated with 10µg/cm^2^ type I collagen (Sigma‐Aldrich) at 37°C, 5% CO_2_. For each experiment, the cells were cultured for 96 hours. For time course experiment, cells were cultured on soft and stiff gel. The medium was collected after 48, 96 and 144 hours. Cell culture medium was collected after each experiment into sterile Eppendorf tubes and centrifuged (*160g*, 10 minutes).

### 2.5 Animals

Twelve‐week‐old male homozygous Itga2^tm1.1Tkun/tm1.1Tkun^ and wild‐type mice were obtained by breeding heterozygous B6.Cg‐Itga2^tm1.1Tkun^/J mice purchased from the Jackson Laboratory (#018921). The mice were housed at germ‐free conditions on a 12:12‐h light‐dark cycle, with free access to commercial food pellet and tap water ad libitum. All animal procedures were approved by the Local Commission of Ethics in Lodz.

### 2.6 Reagent preparation

Several inhibitors were used in this experiment: TC‐I 15 (potent α2β1 integrin inhibitor), AZM 475271 (Src kinase inhibitor) and FAK inhibitor 14 (FAK kinase inhibitor) (Tocris). Both TC‐I 15 and AZM 475271 were dissolved in DMSO (final concentration of DMSO was 0.001% and 0.002%, respectively), (Sigma‐Aldrich) and used at concentrations of 10^−7^ mol/L and 10^−8^ mol/L. Two control groups were applied: one being an untreated control (CTR) and the other composed of cells incubated with 0.001% or 0.002% DMSO. FAK inhibitor 14 was dissolved in water and used at concentrations of 10^−5^ mol/L, 10^−6^ mol/L and 10^−7^ mol/L and compared with control group. The medium was changed every day.

### 2.7 ITGA2 gene silencing

ITGA2 gene silencing was performed by means of siRNA (Dharmacon™). siRNA (25 µmol/L, final concentration) was transfected into cells by RNAiMAX reagent (Thermo Fisher Scientific) according to the manufacturer's instructions. The cultures were divided into the following groups: intact control (CTR), cells treated with non‐targeting siRNA (NT) or cultures administered with siRNA to silence expression of ITGA2 gene (ITGA2). The treated cells were incubated for six hours; after this time, the culture medium containing siRNA was changed to full culture medium. The level of ITGA2 silencing was measured by qPCR and flow cytometry.

### 2.8 Flow cytometry

Flow cytometry experiments were performed to confirm the presence of α2 and β1 integrin subunits on the membrane of the immortalized human cardiac fibroblast cell line; the aim was to measure the changes in the density of the α2 subunit, which was dependent on the physical properties of the cell culture environment, and to measure the ITGA2 silencing levels. After fixation with BD Cytofix (BD Biosciences, Franklin Lakes, NJ, USA), the cardiac fibroblasts were stained (4°C for 25 minutes) in BD Pharmingen stain buffer (BD Biosciences), with the following antibodies: mouse anti‐integrin alpha 2 antibody [AK7] (FITC) (Abcam) and mouse IgG1 kappa [MOPC‐21] (FITC)—isotype control (Abcam). Samples were analysed using a Becton‐Dickinson FACScan analytical flow cytometer (Becton‐Dickinson).

### 2.9 qPCR

Total RNA was isolated using a minicolumn Total RNA Mini Kit (A&A Biotechnology). The concentration of RNA was measured using a NanoDrop™ One Spectrophotometer (Thermo Fisher Scientific). Following this, 500 ng of RNA was transcribed into cDNA using PrimeScript RT‐PCR Kit (Takara).

Amplification reactions were performed using Universal Probe Library (UPL) (Roche) and RealTime ready Custom Single Assay (Roche) based on TaqMan probes (for *ITGA2, GAPDH, RPLP0* and *Ywhaz*). The reactions were conducted using FastStart Essential Probe Master (Roche) according to the manufacturer's protocol. The reactions were carried out with the following programme: 95°C for 10 minutes followed by 55 cycles of 95°C for 10s, 60°C for 30s and 72°C for 1s, followed by 40°C for 30s. Ribosomal protein 0 (*RPLP0*), tyrosine 3‐monooxygenase/tryptophan 5‐monooxygenase activation protein zeta (*Ywhaz*) and glyceraldehyde‐3‐phosphate dehydrogenase (*GAPDH*) were used as a reference genes. Each reaction was conducted in duplicate and expressed as relative expression calculated in LightCycler^®^ 96 software (Roche).

### 2.10 Enzyme‐Linked Immunosorbent Assay (ELISA)

The IL‐6 levels in cell culture media samples and mouse serum were measured using a specific ELISA kit (E‐EL‐H0102 and E‐EL‐M0044, Elabscience). The sIL‐6 was measured in cell culture media samples and in homogenized heart tissue using MBS266072 and MBS2516331 (MyBioSource) according to the manufacturer's instructions. Following this, heart tissue was homogenized in ice‐cold PBS (0.1 mol/L, pH = 7.4) (tissue weight (g): PBS (mL), volume 1:9) and then centrifuged (5000×g, 5 minutes). Supernatant was collected for measurements. Content of integrin α2β1 receptor and IL‐11 in fibroblasts were measured using E01I0003 (BlueGene Biotech) and E‐EL‐H5022 (Elabscience), respectively. The cells were sonicated for evaluation of α2β1 integrin. Before assay, the samples were appropriately diluted. Absorbance was determined at 450 nm using an EL × 800UV Universal Microplate Reader (BioTek Instruments Inc).

### 2.11 Statistical analysis

Results were presented as mean ± SD. Statistical differences were analysed using the independent two‐sample t test or one‐way ANOVA with Bonferroni post‐test correction. All analyses were performed using Statistica 13.1 software (StatSoft). Results with *P*‐value below .05 were regarded as statistically significant. All experiments were performed in at least four independent replicates.

## 3 RESULTS

### 3.1 Mechanical properties of polyacrylamide gels

**FIGURE 1:**
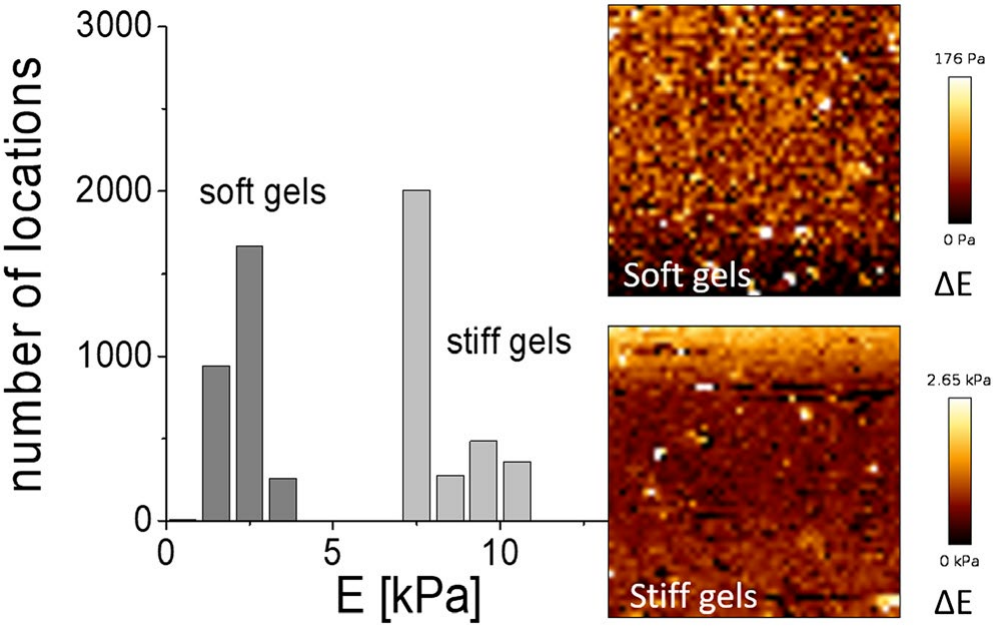
Young's modulus distributions obtained for soft and stiff gels (mean 2.23 ± 0.80 kPa and 8.28 ± 1.06 kPa for soft and stiff gel samples, respectively). Scan size; 50 µm × 50 µm (256 × 256 pixels). Inset: Example images showing the corresponding elasticity maps illustrating the variability of the moduli around the mean value, Δ*E*

The mechanical properties of the prepared soft and stiff gels were compared. Indentation tests performed on both gels found the mean values of the elastic modulus equalled to 2.23 ± 0.8 kPa for the soft gels (*n* = 10) and 8.28 ± 1.06 kPa for the stiff gels (*n* = 10). Variability of stiffness changes, Δ*E*, is presented as the 2D distribution maps, obtained for soft and stiff gels. Results show the degree of gel homogeneity in terms of sample rigidity (Figure 1).

The mechanical properties of the soft materials were determined using an atomic force microscope, which produces a very detailed distribution map of the elastic modulus. The sample is probed with the use of cantilevers ending with a very fine tip. In our study, used OTR4 cantilevers fitted with a tip with a radius of curvature of 15 nm.

To demonstrate that the hydrogels are not uniform at the nanoscale, two types of measurements were conducted. The distributions of Young's modulus (elastic modulus measured during compression) were measured over a scan of 10 × 10 µm. These distributions are characterized by a certain width which may indicate a systematic error in the AFM measurements, but also may reflect the mechanical variability of the hydrogel samples in nanoscale measurements. To illustrate, two images of Young's modulus distribution are shown for a large area of 50 × 50 µm measured with a resolution of 195 nm (Figure 1). The observed variability is a result of the non‐linearity of the polymerization process. Typically, the reported moduli for hydrogels are G’ (shear modulus), as measured by rheometer using a punch with a diameter of 2.5 cm or rarely 0.8 cm. However, averaging the values over a large sample volume does not allow the nanoscale variability of shear modulus to be observed. In AFM, such averaging is performed by increasing the number of measurements and determining Young's modulus from histograms.

### 3.2 ***α2 and β1*** integrin subunits expression

**FIGURE 2:**
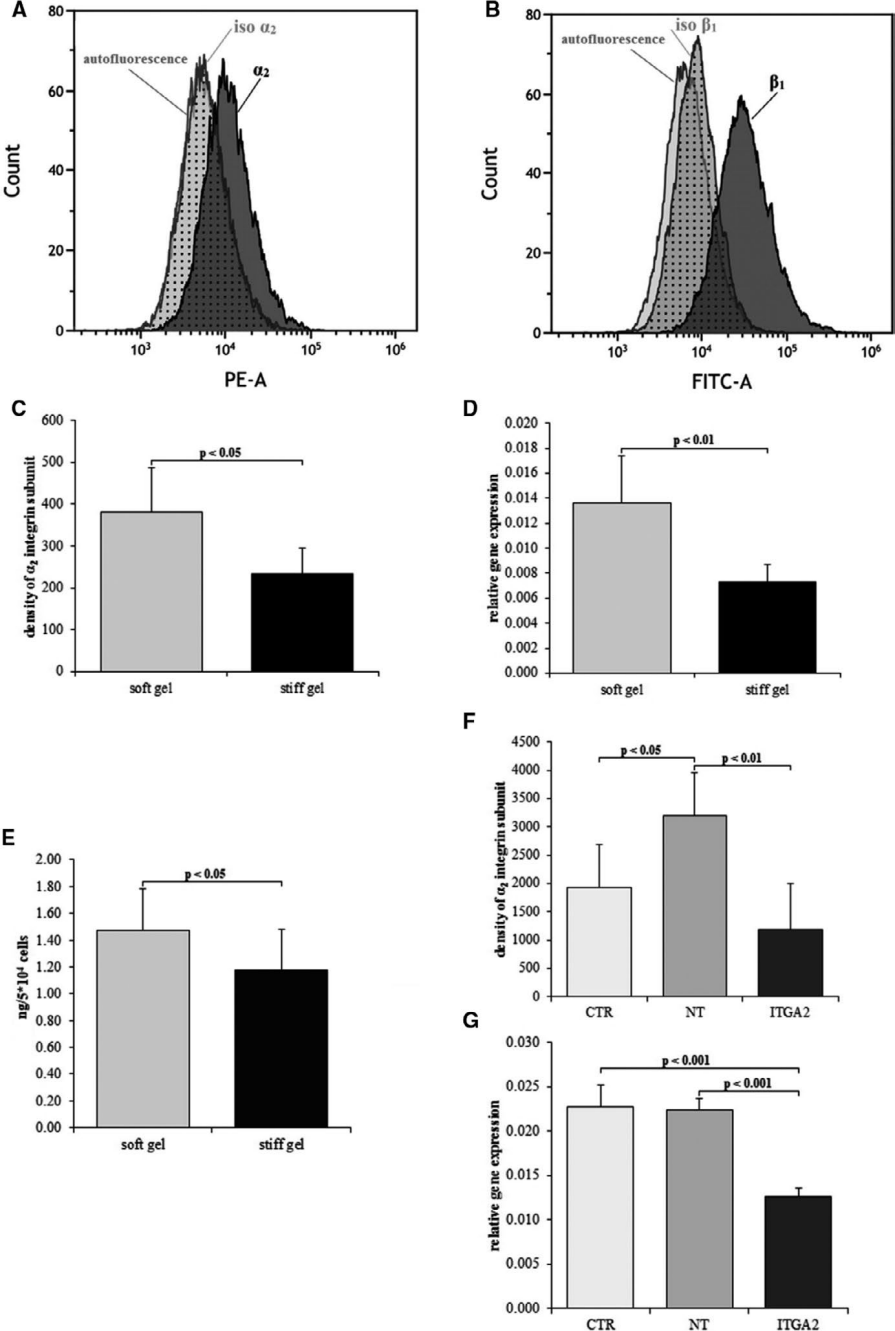
FACS profile showing the expression of α2 integrin subunit (A) and β1 integrin subunit (B) within cardiac fibroblasts compared to isotype control and cellular autofluorescence control. Density of α2 integrin subunit (C) and relative expression of *ITGA2* gene (D), content of integrin α2β1 (E) in cardiac fibroblasts cultured on soft and stiff gels. Density of α2 integrin subunit (F) and relative expression of *ITGA2* gene (G) in cardiac fibroblasts after silencing of α2 integrin subunit (ITGA2), in cells treated with non‐targeting siRNA (NT) and untreated control (CTR). Each value represents a mean ± SD

The expression of α2 and β1 integrin subunits in an immortalized human cardiac fibroblast cell line was evaluated on the protein level by means of flow cytometry. The presence of the subunits was monitored by fluorescence‐activated cell sorting (FACS) (Figure 2A,B). In addition, the levels of α2 and β1 integrin subunits were evaluated and compared with an autofluorescence sample and isotype control. The expression of the α2 integrin subunit was compared between immortalized human cardiac fibroblasts cultured on soft and stiff gels; differences in both mRNA and protein expression were evaluated. The decrease (*P* < .05) in surface rigidity resulted in a 1.63‐fold increase in the density of the α2 integrin subunit (381.8 ± 104 vs. 234.4 ± 60; Figure 2C). A similar effect was observed on the mRNA level (*P* < .01) with a 1.86‐fold relative increase in gene expression on the softer gel (0.0136 ± 0.0037 vs. 0.0073 ± 0.0014; Figure 2D). The level of the integrin α2β1 receptor was evaluated in cells cultured on surfaces with different rigidity. The level of integrin α2β1 was found to be 1.24‐fold higher (*P* < .05) than in cells cultured on stiff substrate (1.47 ± 0.31 ng/ml vs. 1.18 ± 0.3 ng/ml; Figure 2E).

### 3.3 Confirmation of successful transfection

Successful transfection was confirmed at both protein and gene expression levels by flow cytometry and qPCR, respectively. ITGA2 was suppressed by 63% at the protein level (*P* < .01) and by 44% at the mRNA level (*P* < .001) in comparison with non‐targeting siRNA (Figure 2F,G). The ITGA2 level was 38% lower at the protein level compared with non‐treated cells (*P* < .05) and 45% lower at the mRNA level (*P* < .001; Figure 2F,G).

### 3.4 Determination of cytokines (IL‐6 and IL‐11) levels

**FIGURE 3:**
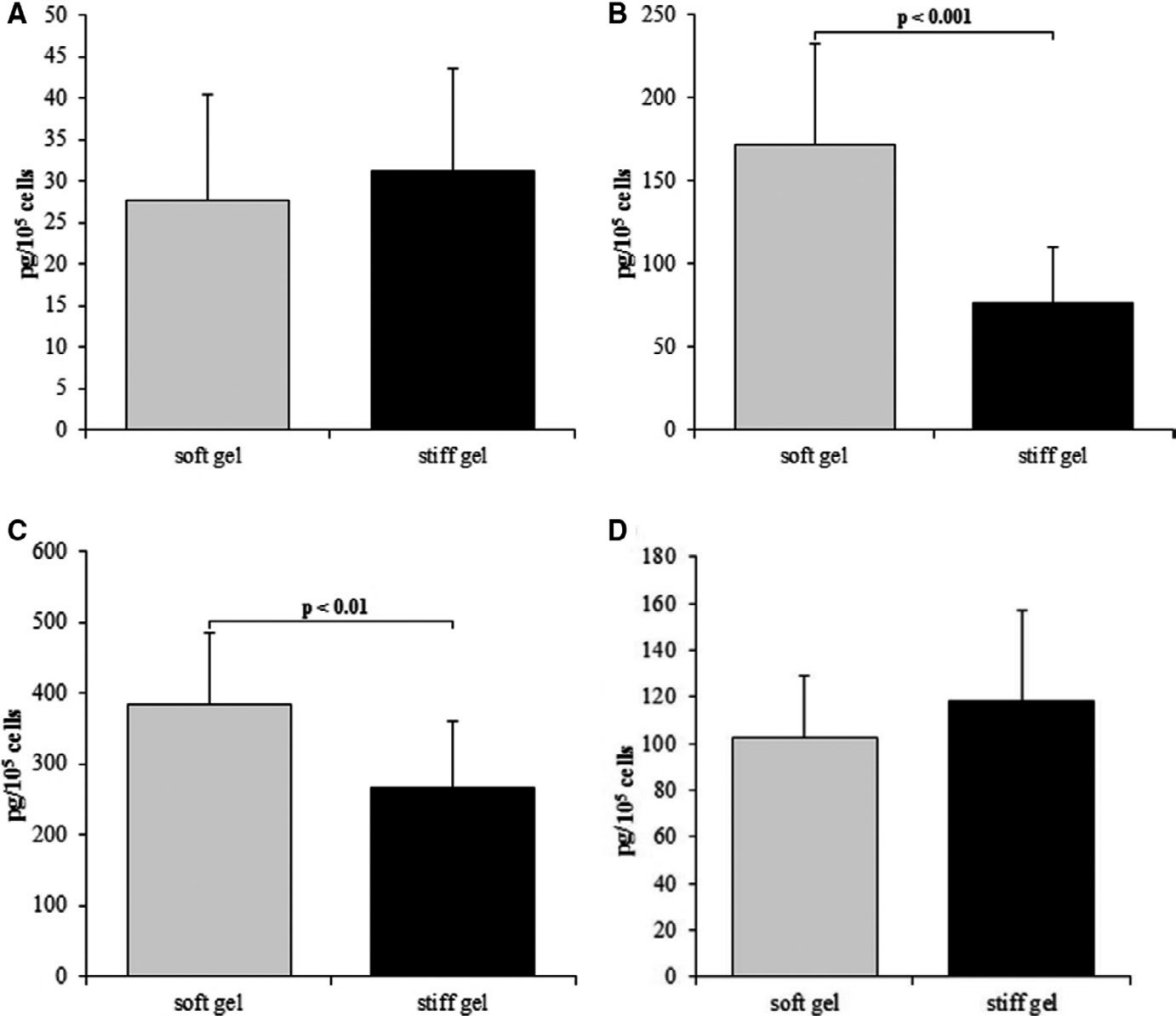
Content of IL‐6 within cardiac fibroblast media cultured on soft and stiff gels after 48 hours (A), 96 hours (B) and 144 hours (C). IL‐11 level within cardiac fibroblast after 96 hours of culture (D). Each value represents a mean ± SD

**FIGURE 4:**
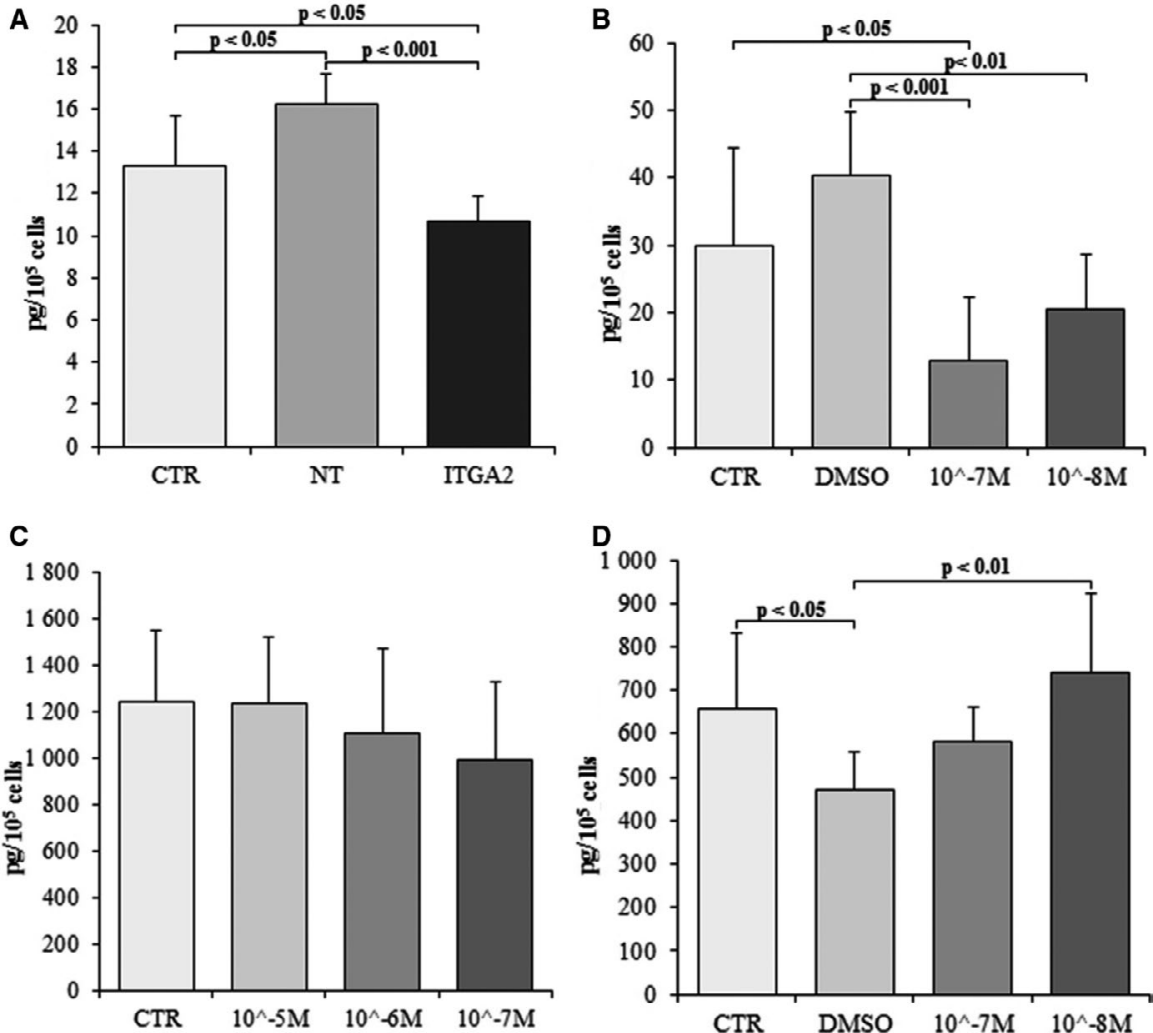
Level of IL‐6 (A) in cardiac fibroblast after silencing of α2 integrin (ITGA2), in cells treated with non‐targeting siRNA (NT) and untreated control (CTR). Effect (B) of TC‐I 15 (α2β1 integrin inhibitor) applied at concentrations of 10^−7^ mol/L and 10^−8^ mol/L, DMSO (TC‐I15 solvent) and untreated control (CTR) on the IL‐6 level in cardiac fibroblasts culture. Effect (C) of FAK 14 inhibitor (FAK kinase inhibitor) applied at concentrations of 10^−5^ mol/L, 10^−6^ mol/L, 10^−7^ mol/L and untreated control (CTR) on the IL‐6 level in cardiac fibroblast culture. Effect (D) of AZM 475 271 (Src kinase inhibitor) applied at concentrations of 10^−7^ mol/L and 10^−8^ mol/L, DMSO (AZM 475271 solvent) and untreated control (CTR) on the IL‐6 level in cardiac fibroblasts culture. Each value represents a mean ± SD

**FIGURE 5:**
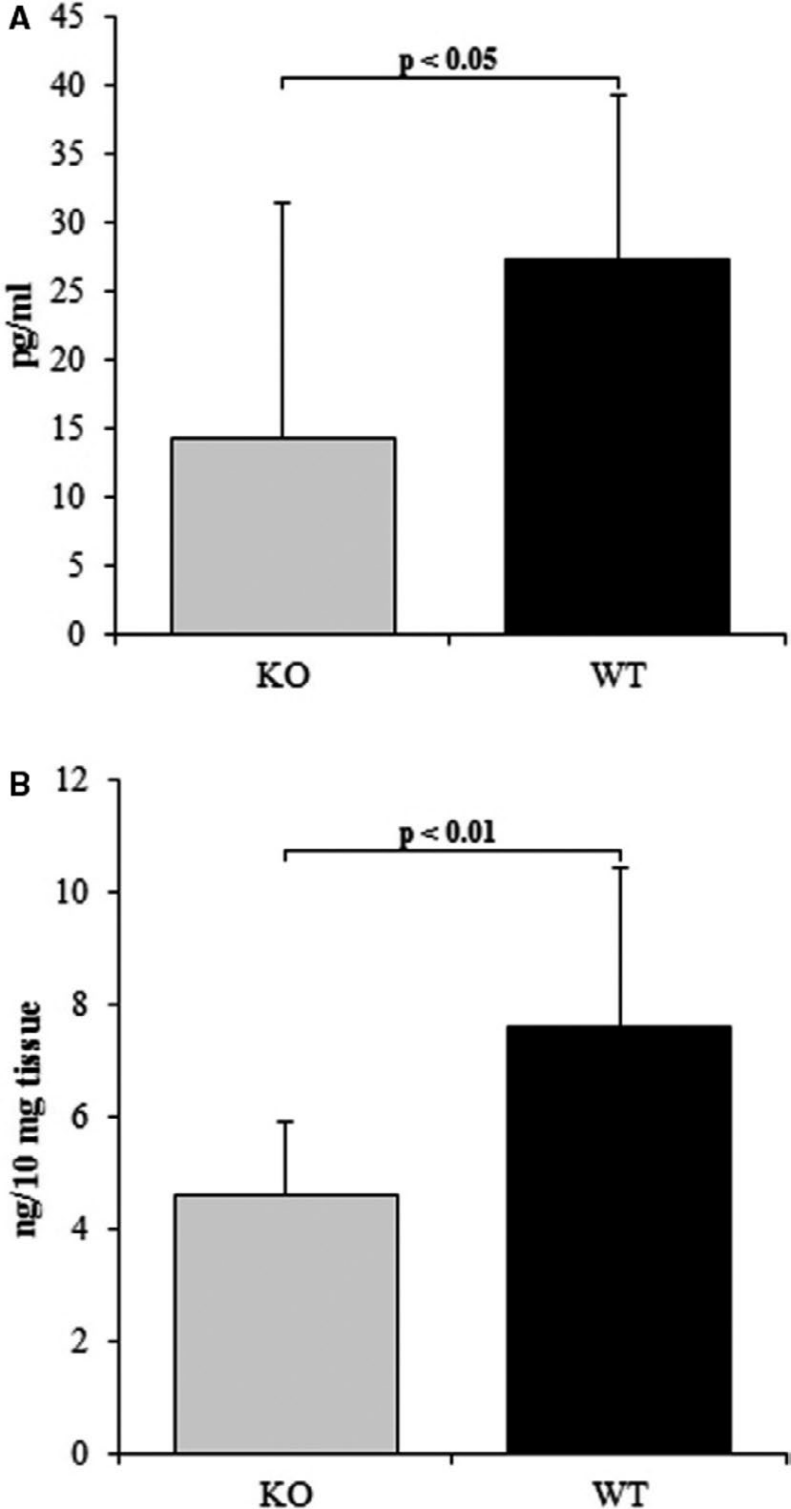
Content of IL‐6 in serum (A) and heart (B) of α2 integrin‐deficient mice (KO) and wild‐type animals (WT). Each value represents a mean ± SD

The concentration of interleukin 6 (IL‐6) was then measured in mouse serum, heart and culture media collected from the performed experiments (Figure 3A‐C). The IL‐6 levels were found to be 1.8‐fold higher in medium from the soft gel than the stiff gel (*P* < .05). The levels of IL‐6 in the medium were higher in the cells cultured on soft gel than those on the stiff gel at two time‐points. The differences between groups increased 2.23‐fold after 96 hours (*P* < .001) and 1.44‐fold after 144 hours (*P* < .01) (Figure 3B,C). No significant differences in IL‐6 (27.7 ± 12.6 pg/mL vs. 31.2 ± 12.4 pg/mL; Figure 3A) after 48 hours as well as in IL‐11 (102.4 ± 26.4 pg/mL vs. 118 ± 39 pg/mL; Figure 3D) after 96 hours were observed between cultures from soft and stiff gels. TC‐I 15 administration decreased IL‐6 level in the culture medium compared to control groups. Statistically significant differences were observed between the groups treated with DMSO and TC‐I 15 at concentrations 10^−7^ mol/L and 10^−8^ mol/L (*P* < .001 and *P* < .01, respectively) and between control (CTR) and 10^−7^ mol/L TC‐I 15 (*P* < .05; Figure 4B). Similarly, the concentration of IL‐6 in α2 integrin‐silenced cells was found to be 1.56‐fold lower than the cultures treated with non‐targeting siRNA (*P* < .001) and 1.25‐fold lower than in non‐treated controls (*P* < .05). A 1.2‐fold change was observed between non‐targeting siRNA control and non‐treated cells (*P* < .05, Figure 4A). These differences illustrate the toxic effects of non‐targeting siRNA. No differences were observed between controls and the groups treated with different concentrations of FAK kinase inhibitor (Figure 4C). Application of Src kinase inhibitor at a concentration 10^−8^ mol/L caused 1.38‐fold increase in IL‐6 levels in comparison with DMSO‐treated group (*P* < .05) (Figure 4D). Lower IL‐6 levels were observed in the DMSO‐treated group than the non‐treated cells (469.32 ± 89.3 pg/mL vs. 658.25 ± 174.9 pg/ml, *P* < .05). No significant change in IL‐6 level was observed between cells treated with 10^−7^ mol/L Src kinase inhibitor compared with DMSO‐treated cells (Figure 4D). The IL‐6 levels were found 1.9‐fold lower in mice without Itga2 than in wild‐type mice (*P* < .05). Moreover, in the heart of knockout mice decreased level of IL‐6 (*P* < .001) comparing with wild‐type mice was reported (Figure 5).

### 3.5 Evaluation of sIL‐6R level

**FIGURE 6:**
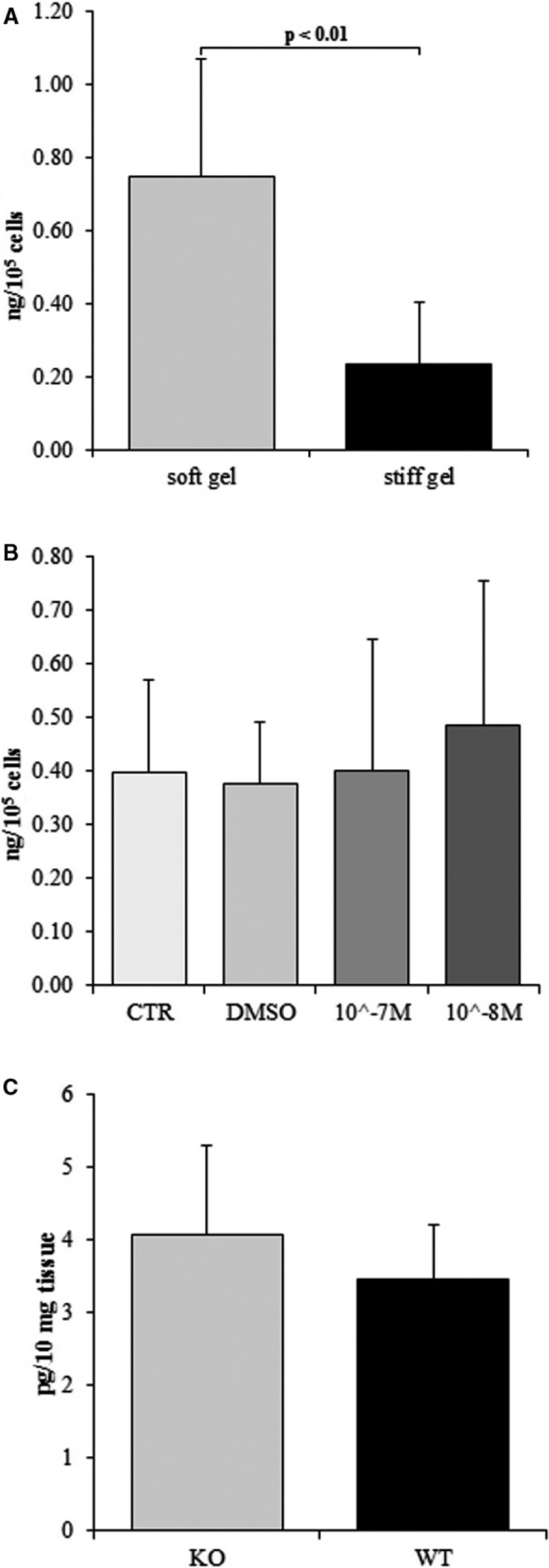
Content of sIL‐6R in cardiac fibroblast media cultured on soft and stiff gels (A). Effect of TC‐I 15 (α2β1 integrin inhibitor) applied at concentrations of 10^−7^ mol/L and 10^−8^ mol/L, DMSO (TC‐I15 solvent) and untreated control (CTR) on the sIL‐6R level in cardiac fibroblast culture (B). Content of sIL‐6R in heart of α2 integrin‐deficient mice (KO) and wild type (WT) in Figure C. Each value represents a mean ± SD

The sIL‐6R levels were found (Figure 6A) to be 3.2‐fold higher in medium from the soft gel than the stiff gel (*P* < .01) Administration of α2β1 integrin inhibitor (TC‐I 15) did not cause any changes between groups (Figure 6B): the sIL‐6R levels in cultures treated with TC‐I 15 at concentrations of 10^−7^ mol/L and 10^−8^ mol/L were not significantly different from those in untreated controls (CTR) and DMSO‐administered cells (DMSO). Furthermore, similar sIL‐6R levels were observed in the hearts of knockout and wild‐type mice (Figure 6C).

## 4 DISCUSSION

Our flow cytometry findings data indicate that both α2 and β1 integrin subunits are expressed on the surface of cardiac fibroblasts (Figure 2A,B). The subunits heterodimerize, forming α2β1 integrin the transmembrane receptor for collagen.^23^ This integrin is also involved in mechanotransduction.^24^ The expression of collagen‐binding integrins such as α1β1, α2β1 and α11β1 on cardiac fibroblast or myofibroblast has been reported earlier,^25^ and α2 integrin has been identified on bone marrow cells, in Wharton's jelly of the umbilical cord cells,^24^ epithelial cells, platelets, megakaryocytes ^26^ and peritoneal mast cells.^27^ To explain the effect of the substrate stiffness on cardiac fibroblasts, polyacrylamide gels of varying stiffness were created.^28^, ^29^ A softer gel with an average Young's modulus of 2.23 ± 0.8 kPa was formed by the addition of 0.06% of bis‐acrylamide; its elasticity corresponds to the rigidity of the cardiac fibroblasts isolated from the left ventricle possessing Young's modulus of 3.38 ± 0.39 kPa.^30^ A more rigid gel with Young's modulus of 8.28 ± 1.06 kPa was also created using 0.1% of bis‐acrylamide; its rigidity markedly exceeded the elasticity of the heart fibroblasts. The stiffness of the applied gels (from 2.23 ± 0.8 kPa to 8.28 ± 1.06 kPa) is related to physiological conditions. Hence, our results suggest that changes in substrate stiffness, within physiological values, may modify the release of IL‐6 by fibroblasts. However, stiffness in pathological conditions with elasticity ranging from 35 to 70 kPa (mimicking the myocardial infarction scar) may induce functional and structural changes in cardiomyocytes, such as deprivation of cardiomyocyte striations and prevention of beating.^31^ As such, there is a need for further investigations of the effect of pathological stiffness on fibroblast structure and function.

The cardiac fibroblasts settled within the soft gel demonstrated increased expression of the α2 integrin subunit gene and higher α2 integrin density on their membrane (Figure 2C). These observations indicate that changes in substrate stiffness may influence the transcription of the α2 integrin gene or may increase the stability of α2 integrin mRNA. In addition, in fibroblasts cultured on the soft gel demonstrated a higher α2β1 integrin count than those on the stiff gel (Figure 2E).

In a previous study, Western blot analysis found α2 integrin expression in dermal fibroblast to be increased on gel containing 0.25% bis‐acrylamide, regarded as a flexible surface, compared to those cultured on a gel with 0.4% bis‐acrylamide.^32^ Interestingly, studies have found the final effect of the substrate stiffness on α2 integrin expression to be dependent on the type of cells; human mesenchymal stem cells with an osteogenic phenotype isolated from Wharton's jelly of the umbilical cord cells cultured on a stiff gel (26.12kPa) demonstrated higher expression than those on a soft gel (1.46 kPa).^24^ Furthermore, the level of integrin α2 observed in mesenchymal stem cells during the induction of osteogenesis was found to be higher in stiffer gels.^33^ This phenomenon was not reported in cultures of mesenchymal cells isolated from bone marrow.^24^

Cardiac fibroblasts are responsible not only for the extracellular matrix metabolism within the heart, and they may also secrete cytokines and other signalling molecules exerting a regulatory influence on cardiomyocytes ^34^ or blood vessels.^35^ Heart fibroblasts are also involved in sensing the mechanical and chemical properties of the environment. The fibroblasts detect mechanical stimuli to respond with adequate synthesis of the extracellular matrix. Mechanical forces are distributed within the heart by the extracellular matrix and conveyed to the cells. Integrin links the extracellular matrix with the cytoskeleton of the cells and participates in mechanotransduction.^36^ Changes in substrate elasticity modify α2 integrin subunit expression (Figure 2C‐E). These results suggest that α2β1 integrin may play an important role in the response of cardiac fibroblasts to modifications of surface stiffness.

The obtained data indicate that IL‐6 concentration was elevated among the fibroblasts cultured on the soft surface (Young's modulus of 2.23 ± 0.8 kPa) than on the stiffer surface (Young's modulus of 8.28 ± 1.06 kPa). The infusion of IL‐6 by minipump induced the conversion of fibroblasts into myofibroblasts. This effect was linked with extensive heart fibrosis and hypertrophy of the left ventricle in a rat model.^37^ Decreased inflammatory reaction and inhibition of heart fibrosis were reported in IL‐6 knockout mice treated with angiotensin‐II.^38^ In addition, González et al report that lack of IL‐6 protects the heart from inflammation, fibrosis and dysfunction;^8^ the cytokine suppresses hypertrophy of the left ventricle, protects cardiac myocytes from apoptosis in pressure‐overloaded hearts and attenuates the area of heart injury.^39^ In addition, IL‐6 level has also been reported to be an independent biomarker for atrial fibrillation,^40^, ^41^ in patients with heart failure displaying significantly higher concentrations.^42^ An elevated level of IL‐6 was also observed in patients after myocardial infarction, with IL‐6 level corresponding with the severity of heart failure.^43^ Low level of IL‐6 on stiff substrate was accompanied by decreased concentration of soluble IL‐6R (sIL‐6R) (Figures 3 and 6). This is additional mechanism lowering IL‐6 dependent effects. TC‐I 15 the α2β1 integrin inhibitor did not influence on sIL‐6R level. Moreover, in α2‐deficient mice the content of sIL‐6R was the same as in wild type. These data suggest that mechanism lowering sIL‐6R in fibroblasts cultured on stiff gel is not dependent on α2β1 integrin (Figure 6). Furthermore, elasticity of substrate did not modified IL‐11 release by cardiac fibroblasts.

Extracellular matrix binding to the cell induces phosphorylation of Src on Thyr527 resulting in inhibition of Src kinase activity.^44^ Similarly, the inhibition of Src kinase by AZM 475 271 in our study was found to elevate IL‐6 content. This observation clearly shows that Src kinase participates in the transmission of the intracellular signal. Focal adhesion kinase (FAK) is not involved in this process.

Several sets of data clearly show that IL‐6 acts as a profibrotic cytokine within the heart.^8^, ^37^, ^38^ IL‐6 concentration was found to be elevated in medium taken from cardiac fibroblasts cultured on the soft gel surface (Figure 3A‐C), and elevated IL‐6 level is linked with higher expression of α2 integrin within cardiac fibroblasts. These observations correspond with earlier data confirming increased collagen content in fibroblast cultures settled on soft gels, and reduced collagen accumulation in cultures were α2β1 integrin was blocked.^10^ In the present study, the level of IL‐6 in medium was found to be lowered by TC‐I 15 inhibition of the α2β1 integrin or siRNA silencing of the α2 integrin subunit (Figure 4A‐B). Although two different methods were used to inhibit integrin α2β1, both models demonstrated lowered IL‐6 concentration in culture medium. These findings suggest that α2β1 integrin may be involved in the regulation of IL‐6 release by cardiac fibroblasts; however, this signal is transmitted by Src kinase (Figure 4D). Moreover, the data obtained on cell cultures are supported by results derived from Itga2^tm1.1Tkun/tm1.1Tkun^ mice. Thus, markedly lower level of IL‐6 in both serum and heart was reported in mice without integrin α2 comparing to wild‐type animals. The integrin α2β1 is responsible for down‐regulation of IL‐6 in both human cardiac fibroblasts (in vitro) and in mice (in vivo).

Previous studies have noted that the α2β1 integrin may play a role in the regulation of the secretion of some cytokines by immune cells. McCall‐Culbreath et al report that mast cells activated by opsonization with immune complex *Listeria Monocytogenes* release IL‐6 in a manner dependent on integrin α2β1 but independent of IgE.^27^ Moreover, in CD4 + T cells, α2β1 integrin‐dependent adhesion to collagen increased secretion of interferon‐γ.^33^

Conclusion. Integrin α2β1 expression was found to be elevated in cardiac fibroblasts cultured on a soft substrate. This phenomenon was found to correspond with increased levels of IL‐6 in the medium. Furthermore, our data suggest that integrin α2β1 exerts a regulatory effect on IL‐6 release by cardiac fibroblasts. The signal is transmitted by Src kinase. Elastic properties of a substrate influence the release of sIL‐6R by cardiac fibroblasts. Hence, in a ‘soft’ environment, action of IL‐6 is not only determined by its increased concentration but is potentialized by higher concentrations of sIL‐6R. The level of sIL‐6R is not dependent on α2β1 integrin. All in all, integrin α2β1 could be considered as a target for anti‐inflammatory therapy associated with heart conditions.

## CONFLICT OF INTERESTS

The authors declare that there is no conflict of interests.

## AUTHOR CONTRIBUTION

**Małgorzata Gałdyszyńska:** Conceptualization (lead); Formal analysis (lead); Investigation (lead); Methodology (lead); Visualization (lead); Writing‐original draft (lead); Writing‐review & editing (lead). **Justyna Bobrowska:** Investigation (supporting); Methodology (supporting); Writing‐review & editing (supporting). **Małgorzata Lekka:** Formal analysis (lead); Investigation (supporting); Methodology (supporting); Supervision (supporting); Validation (supporting); Visualization (supporting); Writing‐original draft (supporting); Writing‐review & editing (supporting). **Paulina Radwańska:** Investigation (supporting); Methodology (supporting); Writing‐review & editing (supporting). **Lucyna Piera:** Investigation (supporting); Methodology (supporting); Writing‐review & editing (supporting). **Jacek Szymański:** Investigation (supporting); Methodology (supporting); Visualization (supporting); Writing‐review & editing (supporting). **Jacek Drobnik:** Conceptualization (lead); Formal analysis (lead); Funding acquisition (lead); Investigation (lead); Methodology (lead); Project administration (lead); Supervision (lead); Validation (lead); Visualization (lead); Writing‐original draft (lead); Writing‐review & editing (lead).

## Data Availability

The data that support the findings of this study are available from the corresponding author upon reasonable request.
